# Exploring pathogen population density as a metric for understanding post-COVID infectious disease surges

**DOI:** 10.3389/fimmu.2024.1459628

**Published:** 2024-10-03

**Authors:** Luwen Zhang

**Affiliations:** School of Biological Sciences, Nebraska Center for Virology, University of Nebraska, Lincoln, NE, United States

**Keywords:** post-COVID surge, herd immunity, COVID-19, pandemic, host resistance, population

## Abstract

After the easing of COVID-19 restrictions, peaks of common infectious diseases surpassed pre-pandemic levels, raising questions about causes and ways to monitor these changes. A proposed measure, the Pathogen Population Density (PPD) score, could help track these shifts. PPD refers to the concentration of infectious agents within a population at a given time and location, serving as a potential indicator of infection levels in susceptible individuals at the population level. It is likely that PPD remains relatively stable within a specific community, as an equilibrium forms between infections and susceptibility. During the pandemic, nonpharmaceutical interventions (NPIs) led to a reduction in infectious diseases, possibly lowering population immunity and decreasing the PPD score. Once NPIs were lifted, the PPD score likely increased sharply due to a larger pool of susceptible individuals, causing more primary infections and stronger recurrent infections, faster transmission, and more severe pathogenic outcomes at the individual level. Monitoring the PPD score over time could help predict when infection peaks will occur. PPD is influenced by factors such as public health strategies, vaccination programs, and the behavior of high-risk individuals. As a quantitative measure, PPD has the potential to serve as a valuable predictive and monitoring tool, helping public health officials anticipate and track changes in infectious disease dynamics. It could be an effective tool for managing future outbreaks or pandemics and serve as a communication tool between scientists and the public to understand the emergence of new disease peaks.

## 1 Introduction

The coronavirus disease (COVID-19) pandemic, caused by the severe acute respiratory syndrome coronavirus 2 (SARS-CoV-2), has had a profound impact on global health. The direct effects of SARS-CoV-2 infection range from asymptomatic to death (1). Additionally, the virus has been associated with long-term health consequences, such as persistent fatigue, cognitive impairment, and cardiovascular complications, collectively known as “long COVID” (2).

In contrast to these direct effects, the pandemic has also had far-reaching indirect consequences on global health. The high mortality rates associated with COVID-19 compelled decision-makers to implement nonpharmaceutical interventions (NPIs) in the absence of an operational vaccine. These public health measures, including lockdowns, social distancing, and mask-wearing, varied widely in their duration, severity, and range across different regions. While these NPIs successfully curbed SARS-CoV-2 transmission (3–5), they also led to a significant decline in normally circulating infections causing known diseases such as influenza, respiratory syncytial virus (RSV), and pneumonia. This decline in known infectious particles during the NPI period likely induced a growing group of susceptible individuals, with the size of this group depending on the specific NPI rules implemented and their duration.

Once COVID19 vaccination became widely available and NPI measures were relaxed, the world witnessed a notable increase in infectious disease cases. This surge, more pronounced than pre-pandemic levels, included viral infections such as RSV, influenza, measles, and more recently, pertussis (whooping cough) and acute mastoiditis (6–13). In addition, disruptions to healthcare services, delays in routine vaccinations, and reduced access to essential medicines have contributed to the overall burden on global health. Moreover, the pandemic has exacerbated existing health disparities, disproportionately affected vulnerable populations, and widened the gap in health outcomes. This phenomenon has puzzled both the scientific community and the public, creating an urgent need for a comprehensive and accessible explanation. The central question that arises is: what is responsible for the higher disease events compared to pre-pandemic levels?

Several known factors can partially explain this increase, including suboptimal vaccination rates due to increased vaccine hesitancy, disruptions to routine immunization programs, an increased pool of susceptible individuals—particularly newborns and young children who lacked exposure to common pathogens during the NPI period—varying levels of NPI implementation, and gaps in public health strategies during the transition from pandemic response to normal operations.

However, these explanations may not fully account for the observed surge. The concept of “immune debt” emerged to explain this increase, suggesting that reduced exposure to common pathogens during the pandemic led to a decline in population immunity, making people more susceptible to infections once restrictions were lifted (7). However, this term has been met with controversy, as critics argue it oversimplifies complex immunological processes and may inadvertently discourage protective behaviors (14). The contentious nature of this concept underscores the challenge of communicating nuanced scientific ideas to the public during a health crisis. Moving forward, it is essential to refine our explanations, balancing scientific accuracy with clear public communication, to better prepare for and respond to future public health challenges. This paper proposes the concept of Pathogen Population Density (PPD) as a potential predictive and monitoring tool that could help bridge the gap between existing explanations and observed post-pandemic disease patterns.

## 2 The pathogen population sensity hypothesis

### 2.1 Definition of the PPD

The concept of PPD refers to the average concentration of a specific infectious agent within a defined human population at a given time. This metric considers both the total number of pathogen units and the proportion of infected individuals, potentially offering new insights into disease risk assessment and public health interventions.

PPD can be measured as the product of two factors: 1) Prevalence of infection within a population: This is the proportion of infected individuals, which can be determined through hospital reports or population-wide testing or sentinel surveillance. 2) Average pathogen load per infected individual: For some pathogens, it may be possible to directly measure pathogen loads in clinical samples (e.g., viral load in blood or respiratory secretions) from a representative sample of infected individuals. Mathematically, PPD can be expressed as: PPD = (Number of infected individuals/Total population) × Average pathogen load per infected individual.

Other than the direct measurement, PPD may be determined or modified through a combination of other factors: 1) Environmental Sampling: For certain pathogens, environmental surveillance (e.g., wastewater testing) might offer an indirect measure of pathogen prevalence. 2) Serological Surveys: While not a direct measure of current PPD, antibody testing can provide information about past infection rates in a population.

### 2.2 Prediction potential of infectious disease activity

Significant changes in PPD can indicate shifts in disease dynamics: 1) A sudden drop in PPD, often due to public health measures, may reduce immediate infection risk but could increase population susceptibility over time. 2) Conversely, higher PPD may lead to more frequent infections, with larger pathogen doses potentially causing more severe primary infections in immunologically naïve populations. Increased PPD could also accelerate the spread of infections, resulting in rapid and intense outbreaks. Therefore, PPD might serve as an early warning system for public health officials, enabling them to anticipate outbreaks before they become clinically apparent. Its measurements may be particularly valuable during 1) Transitions in public health measures, such as the relaxation of NPIs. 2) Monitoring potential resurgences of controlled diseases. 3) Tracking seasonal variations in infectious disease prevalence.

In addition, the quantitative nature of PPD allows for temporal and geographical comparisons, facilitating: 1) Tracking PPD over time to identify trends and predict outbreaks, enabling proactive interventions. 2) Comparing PPD across regions to identify hotspots and allocate resources effectively. 3) Offering value in densely populated areas, regions with low vaccination rates, or where traditional surveillance is challenging. PPD may provide a standardized metric for infection risk, offering a comprehensive tool for monitoring and predicting infectious disease dynamics in various contexts. By providing a quantifiable measure of pathogen presence in a population, PPD could enhance our ability to forecast disease trends and evaluate the effectiveness of public health interventions in real-time.

### 2.3 Critical criteria for effectiveness

For PPD to be effective, it must meet several criteria: 1) Ease of measurement and interpretation. 2) Reliability and consistency across different pathogens and populations. 3) Sensitivity to detect meaningful changes in infection risk. 4) Specificity to distinguish between different pathogens or strains. 5) Economic value for widespread implementation. Through rigorous examination, we can determine whether PPD can become a valuable index to our arsenal of public health metrics, offering unprecedented capabilities for comparison and analysis across diverse scenarios and populations.

## 3 Can PPD monitor and predict the recent surge in infectious diseases after COVID19?

### 3.1 Primary infection with higher pathogen population density

Infection can be divided into primary and recurrent infections based on the sequence of exposure to the pathogen at individual level. Primary infections are the first encounter and often more severe, while recurrent infections are subsequent encounters with generally milder symptoms due to pre-existing immunity in general (15–17). Recurrent infection can also be described as secondary, reinfection, or breakthrough infection.

In general, primary infections may generate more pathogens than secondary or recurrent infections due to several factors: 1) The lack of pre-existing immunity allows the pathogen to replicate more easily and reach higher levels in the host; 2) Primary infections often last longer, giving the pathogen more time to replicate and produce more infectious particles; and 3) Hosts may shed more pathogens during primary infections due to the lack of immunity and longer infection duration. However, the relationship between infection type and infectious pathogen can vary depending on the specific pathogen and host factors.

It is crucial to distinguish between sources of infection and types of infection. Primary sources of infection are the original reservoirs of pathogens, such as environmental sources or specific host populations, while secondary sources are infected individuals or contaminated objects that spread the pathogen after acquiring it from the primary source. Importantly, sources can evolve; a secondary source may become a primary source if the original primary source is eliminated or controlled. This differs from the concept of primary and secondary infections, which refer to an individual’s first encounter with a pathogen and subsequent infections, respectively. Finally, targeting primary sources, such as through vaccination, can reduce infection spread. After a pandemic, a robust vaccination strategy is essential to prevent new infection peaks and ensure effective public health interventions.

Population immunity, also known as herd immunity, is a key concept in infectious disease epidemiology that refers to the indirect protection from an infectious disease that occurs when a significant portion of a population becomes immune to the pathogen. This immunity can be acquired through vaccination or prior exposure to the pathogen (18, 19). The threshold for achieving population immunity varies depending on the infectiousness of the pathogen, with highly contagious diseases requiring a higher percentage of immune individuals (20). While adaptive immunity can explain the molecular basis of population immunity against infectious diseases, it may not always be the most effective way to communicate these concepts to the public. The term “immune shortage” could potentially serve as a more accessible and intuitive way to convey the idea of reduced population immunity, especially in the context of the COVID-19 pandemic. By emphasizing the “shortage” aspect, it highlights the potential consequences of a lack of exposure to common pathogens due to public health measures (14).

### 3.2 Infectious diseases in different age group

To dissect the most recent sudden surge and higher-than-pre-pandemic levels of severity of infectious diseases cases, we need to separate our population into roughly two categories: adult and pediatric populations.

#### 3.2.1 Adults

Due to immune waning and immunosenescence, adult population, especially old individuals, will have reduced immunity against common pathogens, which can be further compounded by reduced exposure to pathogens, delayed or missed vaccinations, comorbidities, frailty, and the mental health impacts of isolation and stress during lockdowns. Elder population is most susceptible to pathogens among adults. When in relaxed contact patterns (removal of NPIs), adult population are encountering common pathogens, it can lead to:

##### 3.2.1.1 More severe recurrent infections

**Figure 1:**
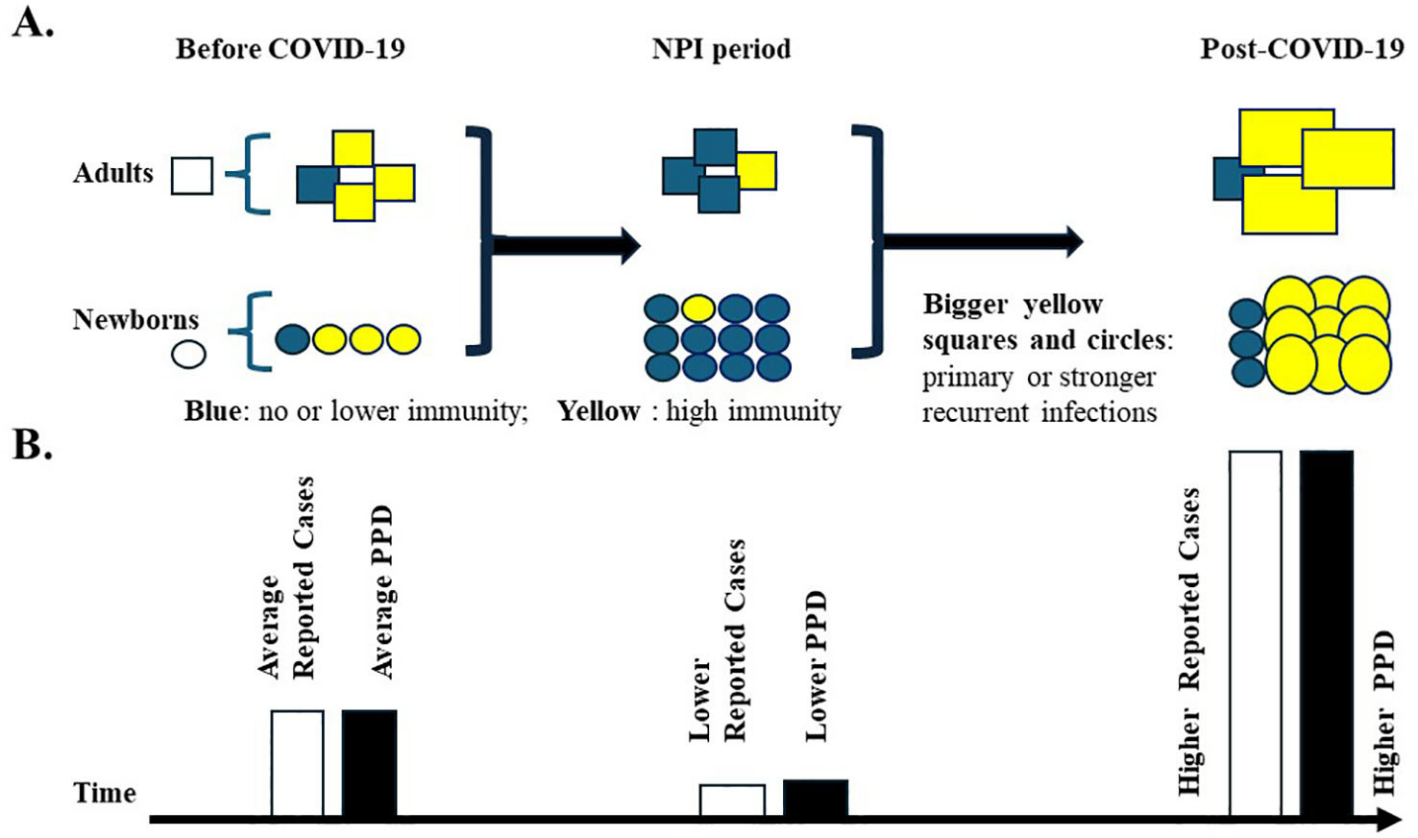
High population pathogen density during the COVID-19 pandemic. **(A)** Susceptible population to common pathogens during the pandemic. Three periods have been classified: Before COVID-19, NPI period, and post-COVID-19. Squares represent adults; Circles represent newborns. Yellow fillings indicate individuals with high immunity against pathogens; blue fillings indicate individuals with no or low immunity against pathogens. The increase in the newborn population is indicated by more circles during the rough pandemic NPI period (2020-2022). The population immunity is set up to be the same before and after the pandemic. Bigger squares and circles with yellow fillings represent primary or stronger recurrent infections of pathogens, which would lead to higher PPD levels. **(B)** Reported infectious disease cases and predicted pathogen population density (PPD) before and after the pandemic. Reported infectious disease cases are indicated by open bars, and hypothetical PPD is indicated by solid bars. The bar heights indicate the putative numbers or values. Average, low, and high values are indicated. Primary infections with higher PPD may lead to more severe symptoms, increased hospitalizations, and higher reported case numbers, especially among children lacking prior immunity.

People who have been exposed previously may still experience reinfection, but the resulting pathogen replication might be increased, especially for pathogens that rely heavily on a strong immune response for control. The pathogen might replicate at a higher rate and for a longer duration compared to a normal reinfection immune response (**Figure 1A**) (21–23).

##### 3.2.1.2 A surge of primary infections

Individuals who have not encountered the pathogen before will be susceptible to a primary infection more easily because of reduced population immunity (**Figure 1A**).

With a larger pool of susceptible adults, these pathogens can cause more primary and/or stronger recurrent infections that readily replicate and spread, resulting in a rapid increase in cases and higher PPD quantitative values compared to pre-pandemic periods. Of note, common pathogens may cause few or no symptoms in adults, leading to an underestimation of infection status.

#### 3.2.2 Pediatric populations

Pandemic prevention measures have left newborns and young children without immunity to common pathogens. This population segment has two key considerations:

##### 3.2.2.1 Higher susceptibility among newborns

Although newborn babies receive some temporary passive immunity from their mothers, it does not cover all pathogens. As a result, newborns born during the NPI period are more susceptible to infections once NPIs are removed. In the US only, the number of live births from 2020 to 2022 is more than nine million (24), contributing to a larger pool of susceptible individuals within the population.

##### 3.2.2.2 Higher pathogen population density

As described above, the surrounding PPDs generated by adult populations might be higher than those before the pandemic. Also, the primary infection of the higher susceptible pediatric population itself may push the PPD to another level (**Figure 1B**). Therefore, the susceptible pediatric population might be exposed to high doses of pathogens after the pandemic.

At the individual level, higher pathogen doses during primary infection can lead to more severe symptoms in many cases. The pediatric population might experience more severe primary infection symptoms compared to pre-pandemic times, which may lead to more hospital visits and reports to health authorities (**Figure 1B**). The PPD concept might best help understand the new surge after the NPIs in the pediatric population.

### 3.3 The impact of NPI duration, severity, and timing on pathogen circulation and PPD

Notably, the duration, severity, and timing of NPI implementation are crucial factors in understanding the consequences for pathogen circulation and their impact on PPD. These factors interact in complex ways to influence both immediate and long-term disease dynamics:

#### 3.3.1 Duration of NPIs

Short-term NPIs may temporarily reduce pathogen circulation, leading to a brief decrease in PPD without significantly impacting long-term population immunity. However, prolonged NPIs can sustain a reduction in PPD, potentially increasing the pool of susceptible individuals over time.

#### 3.3.2 Severity of NPIs

Less stringent NPIs (e.g., mask-wearing in public spaces) may moderately reduce pathogen circulation and PPD, while allowing some level of continued exposure and immune stimulation. In contrast, strict NPIs (e.g., lockdowns, school closures) can dramatically reduce PPD across multiple pathogens, potentially leading to more substantial impacts on population immunity and increasing the risk of disease resurgence once NPIs are relaxed.

#### 3.3.3 Timing of NPI implementation

Implementing NPIs during peak seasons for certain pathogens (e.g., influenza in winter) may have more pronounced effects on PPD and subsequent disease patterns. Additionally, the timing of NPIs in relation to natural cycles of waning immunity in the population can affect the size of the susceptible pool and the potential for disease resurgence.

#### 3.3.4 Pathogen-specific considerations

Different pathogens may be affected differently by NPIs, depending on their transmission routes and environmental stability. The impact on PPD may vary across pathogens, potentially altering the typical balance of circulating pathogens in a population.

#### 3.3.5 Long-term consequences

It is likely that PPD remains relatively stable within a specific community, as an equilibrium forms between infections and susceptibility under normal circumstances. However, a sudden drop in PPD, such as that caused by NPIs, may lead to a “rebound effect” when these measures are relaxed. This effect, characterized by a rapid increase in PPD, may be particularly pronounced for pathogens with shorter immunity duration or those requiring regular boosting of immunity through repeated exposure.

Monitoring PPD throughout the implementation and relaxation of NPIs could provide valuable insights into the dynamic relationship between public health measures and disease risk. This approach could potentially allow for more informed and timely decision-making in managing infectious disease outbreaks.

### 3.4 The off-season effects of certain infectious diseases

It is known that the threshold of pathogen levels needed to trigger an infectious disease outbreak is influenced by several factors, including the minimum infectious dose, population immunity, environmental conditions, host susceptibility, and transmission dynamics (25, 26). PPD can be linked to the seasonality of infections through various interconnected factors. PPD levels may naturally fluctuate with seasons due to environmental conditions, human behavior patterns, and biological cycles of pathogens. These seasonal fluctuations in PPD may offer insights into why certain infections are more prevalent at specific times of the year. The PPD concept suggests that a certain threshold of pathogen concentration might be necessary to trigger an outbreak, and seasonal factors could push PPD over this threshold. For instance, winter crowding might raise PPD for respiratory pathogens, while summer heat could increase PPD for certain vector-borne diseases. Seasonal variations in human immune function and behaviors (like school terms or holiday travel) can also affect susceptibility and contact rates, influencing PPD. Importantly, PPD could help understand off-season outbreaks of typically seasonal diseases if it remains high outside the usual season. By monitoring PPD levels as seasons change, we might improve predictions of seasonal disease patterns and timing of interventions. This approach offers a more nuanced understanding of infection seasonality, potentially enhancing our ability to predict and respond to both regular seasonal outbreaks and unexpected off-season disease events.

### 3.5 Vaccine and infectious disease surge

Vaccine hesitancy contributes significantly to recent surges in infectious diseases (27), directly impacting PPD values. The reluctance or refusal to receive vaccinations has created a pool of susceptible individuals who lack specific immunity against certain pathogens. This increased susceptibility allows pathogens to replicate more efficiently and spread more widely, elevating PPD levels (**Figure 1B**). The COVID-19 pandemic has exacerbated this issue, as the focus on SARS-CoV-2 vaccination efforts has led to a decline in routine childhood vaccinations (28). This decline, coupled with reduced exposure to common pathogens during lockdowns, has likely resulted in lower population immunity and potentially higher PPD once restrictions were lifted. The combination of vaccine hesitancy and reduced pathogen exposure during the pandemic has created a perfect storm for the resurgence of infectious diseases, particularly among pediatric populations, where higher PPD levels may lead to more severe primary infections in the newborns during the NPI periods. Moreover, vaccine hesitancy can create pockets of high PPD within communities, even if overall vaccination rates are high. These localized areas of elevated PPD can serve as reservoirs for pathogens, potentially triggering outbreaks that spread to broader populations. Vaccination remains the most effective way to prevent infections and their potential complications by reducing both individual susceptibility and overall PPD.

### 3.6 SARS-CoV-2 and long-term immune effects

While SARS-CoV-2 infection has been associated with potential long-term immune damage (29–31), it is important to recognize that many other viruses can also cause similar immune dysregulation (32–36). Although SARS-CoV-2-mediated immune damage may contribute to these surges, its role is likely minor compared to the other factors at the population level. Further research is needed to fully understand the long-term immune consequences of COVID-19 and its impact on the resurgence of other infectious diseases.

## 4 Conclusion and challenges

This paper invites critical discussion on the potential of Pathogen Population Density (PPD) as a public health tool. The public health measures associated with the COVID-19 pandemic may have unintentionally led to an immune shortage against common pathogens, subsequently resulting in an increased number of susceptible individuals. More primary and/or stronger recurrent infections would happen, and lead to higher PPD, and the higher PPD could contribute to more infections with severe symptoms and increased reports to health authorities, particularly among children and elderly populations. These outbreaks may also occur outside their usual seasonal patterns as societies return to normalcy. It is tempting to speculate that PPD maintains a balanced and relatively stable value in a community under normal circumstances. A sudden drop in PPD may pave the way for a later surge of infectious diseases, characterized by a higher-than-normal PPD (**Figure 1B**). Further research is needed to refine the concept, develop standardized measurement protocols, and establish its validity across various epidemiological contexts. Testing the PPD hypothesis in daycare centers and nursing homes could provide valuable insights into its validity and practical applications. These environments are particularly suitable for such studies due to their semi-controlled nature and the presence of potentially vulnerable populations.

### 4.1 Challenges

#### 4.1.1 Pathogen-specific factors

Each pathogen’s unique characteristics (e.g., environmental stability, mode of transmission) will affect how PPD manifests and can be measured.

#### 4.1.2 Environmental influences

Factors like temperature, humidity, and air quality may affect PPD differently for various pathogens.

#### 4.1.3 Measurement standardization

Developing standardized PPD measurement techniques across different pathogens will be challenging but may be necessary for comparative studies.

#### 4.1.4 Integration with existing surveillance

PPD monitoring should complement, not replace, traditional disease surveillance methods.

By considering PPD in the context of specific diseases, we can develop a more nuanced understanding of post-pandemic infectious disease dynamics. The PPD approach could potentially improve our ability to predict, monitor, and respond to disease outbreaks across a spectrum of pathogens. As a quantitative and potentially standardizable measure, PPD offers a unique opportunity to develop early warning systems and to track the effectiveness of public health interventions in real-time, potentially revolutionizing our approach to infectious disease management. Also, PPD may serve as an effective communication tool for both the scientific community and the public to understand the sudden surge of infectious diseases after NPIs. As we navigate the post-COVID era, we must learn from the pandemic experience and apply those lessons to strengthen our public health systems.

## Data Availability

Publicly available datasets were analyzed in this study. This data can be found here: PubMed.
